# Supplementary material for: Expression, purification and preliminary pharmacological characterization of the Plasmodium falciparum membrane-bound pyrophosphatase type 1
Source: PLoS One. 2025 May 27;20(5):e0322756. doi: 10.1371/journal.pone.0322756 (PMC12111632; doi:10.1371/journal.pone.0322756)
Supplement: S1 File — S1 Fig. BN PAGE of PfPPase-VP1 in three detergents. The BN PAGE showing the ratio of PfPPase-VP1 dimer to monomer in DDM, GDN, and LMNG. The samples were obtained from the same expression batch, and the table presents the percentage of monomeric PfPPase-VP1, measured using ImageJ based on band intensity (Area (U)). S2 Fig. Removal of GFP using TEV protease. (A) Coomassie-stained SDS PAGE showing PfPPase-VP1_GFP incubated with TEV under different conditions (overnight at 4 °C; 2 h at RT and then overnight at 4 °C). The band at 25 kDa is TEV protease. (B) In-gel fluorescence of PfPPase-VP1 GFP incubated with TEV under different conditions, showing that GFP remains uncleaved despite different incubation conditions. S3 Fig. Expression of PfPPase-VP1 from different batches. (A) GFP fluorescence and Coomassie-stained SDS-PAGE showing purified PfPPase-VP1 from three expression batches. (B). CN PAGE showing the purified PfPPase-VP1 dimer (blue star) and monomer (red star). (C) BN PAGE showing the purified PfPPase-VP1 dimer (blue star) and monomer (red star). The percentage of monomer PfPPase-VP1 were measured using ImageJ based on their band intensity (Area(U)). (D). Characterization of the oligomeric states of PfPPase-VP1 using SEC-MALS. The blue, black and red curves represent the MW of PfPPase-VP1 complex with GDN, the MW of GDN and the MW of PfPPase-VP1, respectively.S4 Fig. Salt-bridge interaction between F726 and R543 in TmPPase. (A) Location of the salt-bridge interaction in TmPPase, which stabilizes the TmPPase dimer. (B) Close-up view of two residues (F726 and R543) forming a salt bridge within 3 Å. The distance is shown in yellow dashed line. The TmPPase is shown in cyan, and two residues are shown in purple. S1 Table 1. Inhibitory activity of readily available compounds targeting PfPPase and TmPPase (PDF) [file pone.0322756.s001.pdf]

Supplementary Figures and Table:

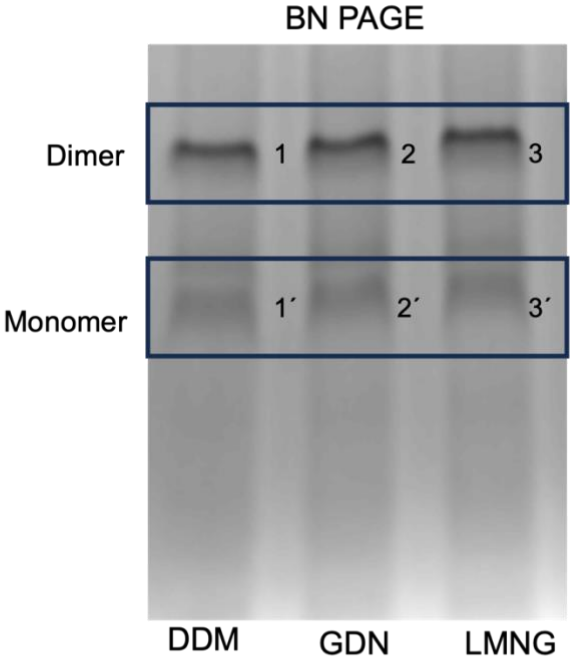

Band No. (n)	1/1´	2/2´	3/3´
Area (U/10000)	1.14/0.5	1.36/0.61	1.41/0.71
Monomer PfPPase-VP1 (%)	30%	31%	34%

S1 Fig. BN PAGE of PfPPase-VP1 in three detergents. The BN PAGE showing the ratio of PfPPase-VP1 dimer to monomer in DDM, GDN, and LMNG. The samples were obtained from the same expression batch, and the table presents the percentage of monomeric PfPPase-VP1, measured using ImageJ based on band intensity (Area (U)).

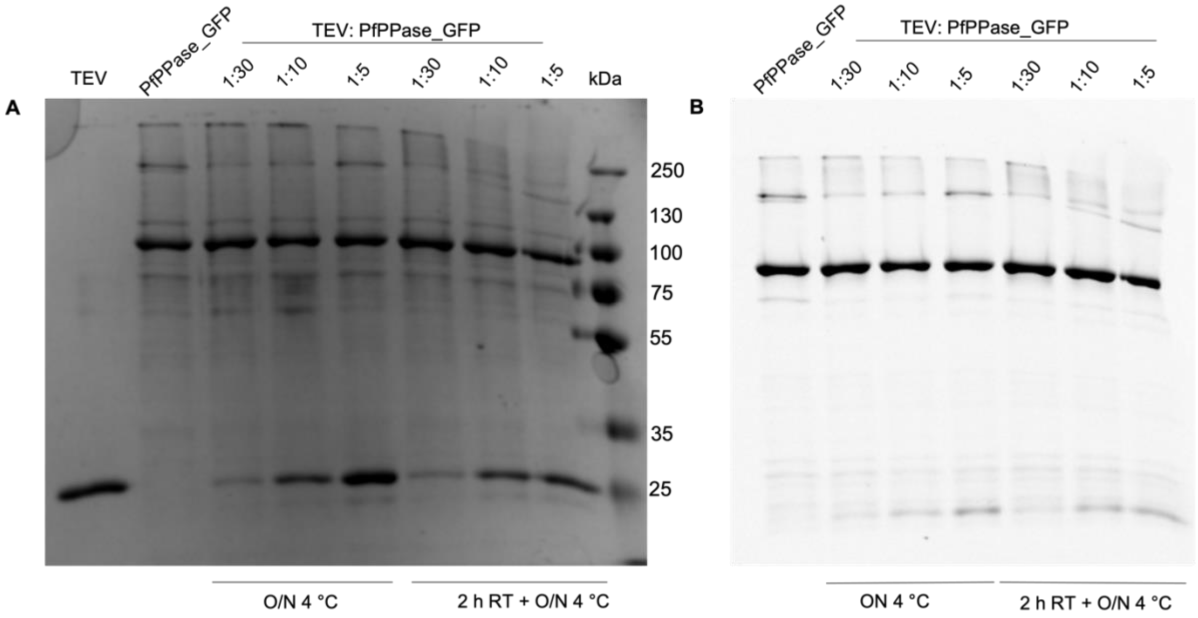

S2 Fig. Removal of GFP using TEV protease. (A) Coomassie-stained SDS PAGE showing PfPPase-VP1_GFP incubated with TEV under different conditions (overnight at 4 °C; 2 h at RT and then overnight at 4 °C). The band at 25 kDa is TEV protease. (B) In-gel fluorescence of PfPPase-VP1 GFP incubated with TEV under different conditions, showing that GFP remains uncleaved despite different incubation conditions.

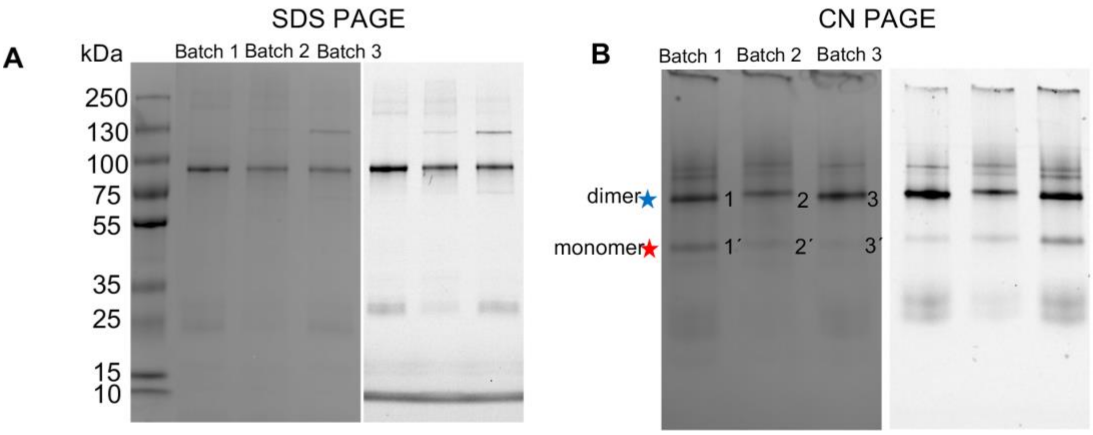

Band No. (n)	1/1´	2/2´	3/3´
Area (U/10000)	1.23/0.43	1.24/0.31	1.25/0.11
Monomer PfPPase-VP1 (%)	26%	20%	8%

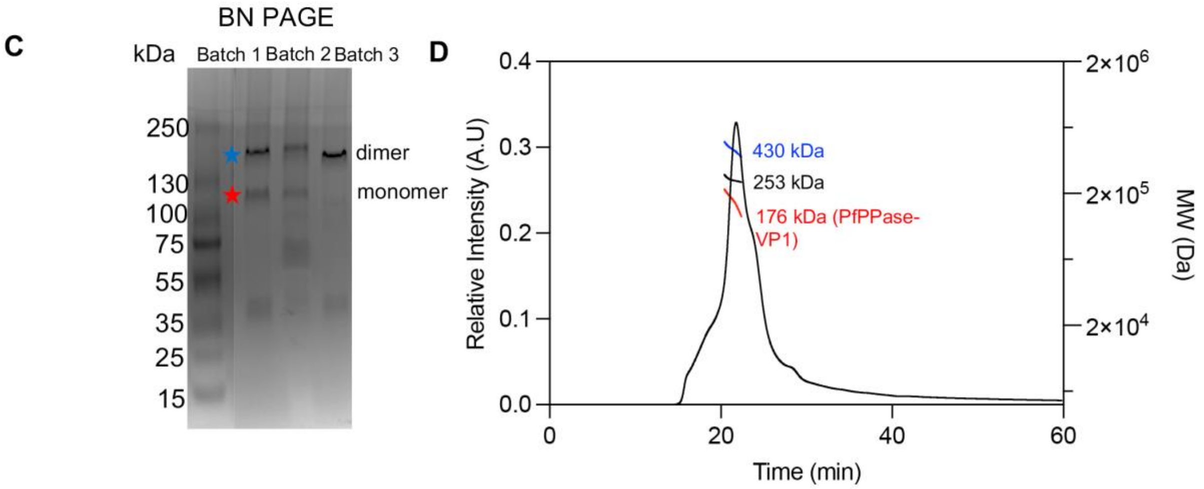

S3 Fig. Expression of PfPPase-VP1 from different batches. (A) GFP fluorescence and Coomassie-stained SDS-PAGE showing purified PfPPase-VP1 from three expression batches. (B). CN PAGE showing the purified PfPPase-VP1 dimer (blue star) and monomer (red star). (C) BN PAGE showing the purified PfPPase-VP1 dimer (blue star) and monomer (red star). The percentage of monomer PfPPase-VP1 were measured using ImageJ based on their band intensity (Area(U)). (D). Characterization of the oligomeric states of PfPPase-VP1 using SEC-MALS. The blue, black and red curves represent the MW of PfPPase-VP1 complex with GDN, the MW of GDN and the MW of PfPPase-VP1, respectively.

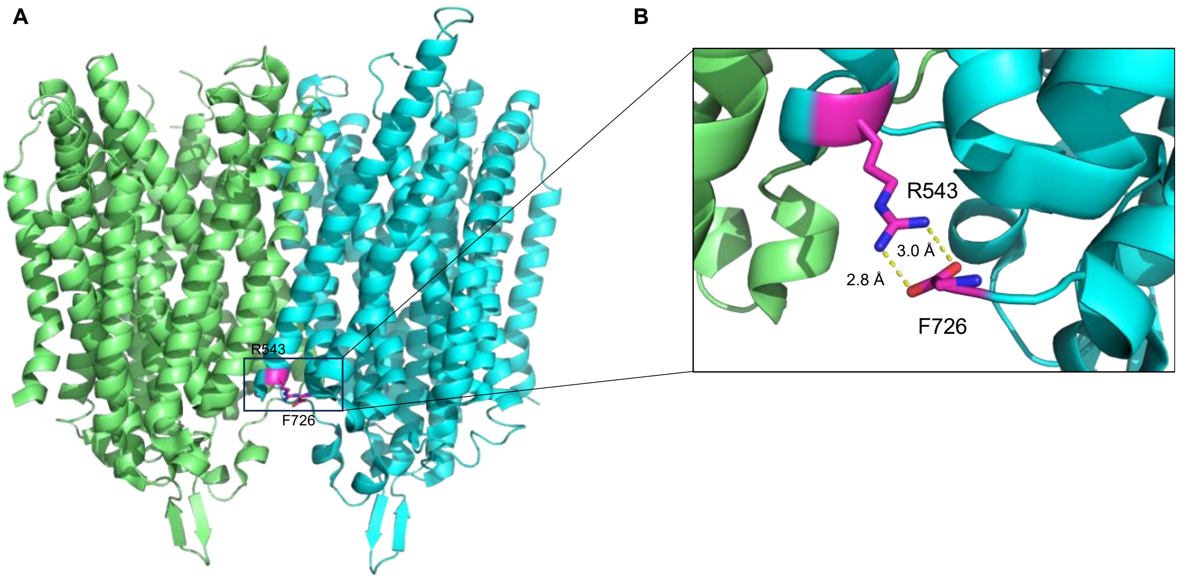

S4 Fig. Salt-bridge interaction between F726 and R543 in TmPPase. (A) Location of the salt-bridge interaction in TmPPase, which stabilizes the TmPPase dimer. (B) Close-up view of two residues (F726 and R543) forming a salt bridge within 3 Å. The distance is shown in yellow dashed line. The TmPPase is shown in cyan, and two residues are shown in purple.

S1 Table 1. Inhibitory activity of readily available compounds targeting PfPPase and TmPPase

Compound	Structure	Scaffold	IC_{50} (TmPPase,μM)	IC_{50} (PfPPase,μM)	Vendor/Reference
22	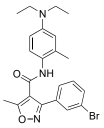	Isoxazole	Inactive	Inactive	ChemDiv
23	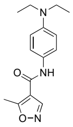	Isoxazole	Inactive	Inactive	MayBridge
24	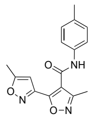	Isoxazole	Inactive	Inactive	MayBridge
25	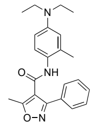	Isoxazole	Inactive	Inactive	MayBridge
26	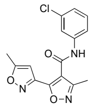	Isoxazole	Inactive	Inactive	MayBridge
27	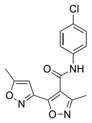	Isoxazole	Inactive	Inactive	MayBridge
28	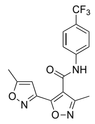	Isoxazole	Inactive	Inactive	MayBridge
29	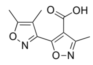	Isoxazole	Inactive	Inactive	In-house synthesis [38]
30	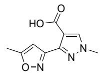	Isoxazole	Inactive	Inactive	In-house synthesis [38]
31	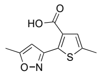	Isoxazole	Inactive	Inactive	Maybridge

32	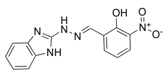	Isoxazole	Inactive	Inactive	ChemBridge
33	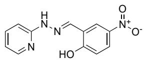	Isoxazole	Inactive	Inactive	ChemBridge
34	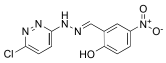	Isoxazole	Inactive	Inactive	ChemBridge
35	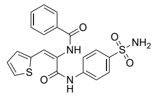	Isoxazole	Inactive	Inactive	ChemBridge
