# Supplementary material for: Expression, purification and preliminary pharmacological characterization of the Plasmodium falciparum membrane-bound pyrophosphatase type 1
Source: PLoS One. 2025 May 27;20(5):e0322756. doi: 10.1371/journal.pone.0322756 (PMC12111632; doi:10.1371/journal.pone.0322756)
Supplement: S1 Raw Images — (PDF) [file pone.0322756.s002.pdf]

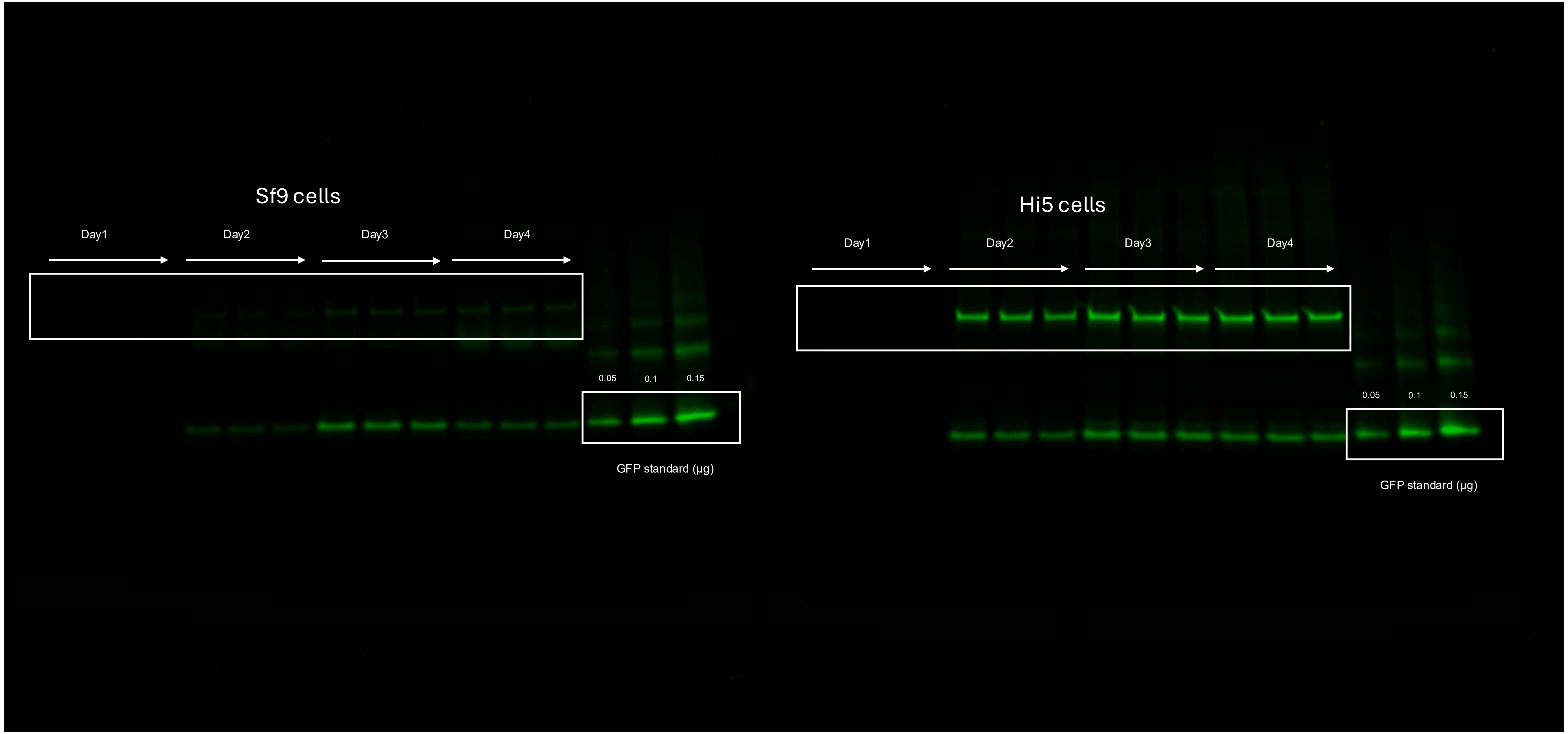

Fig 1. the raw image

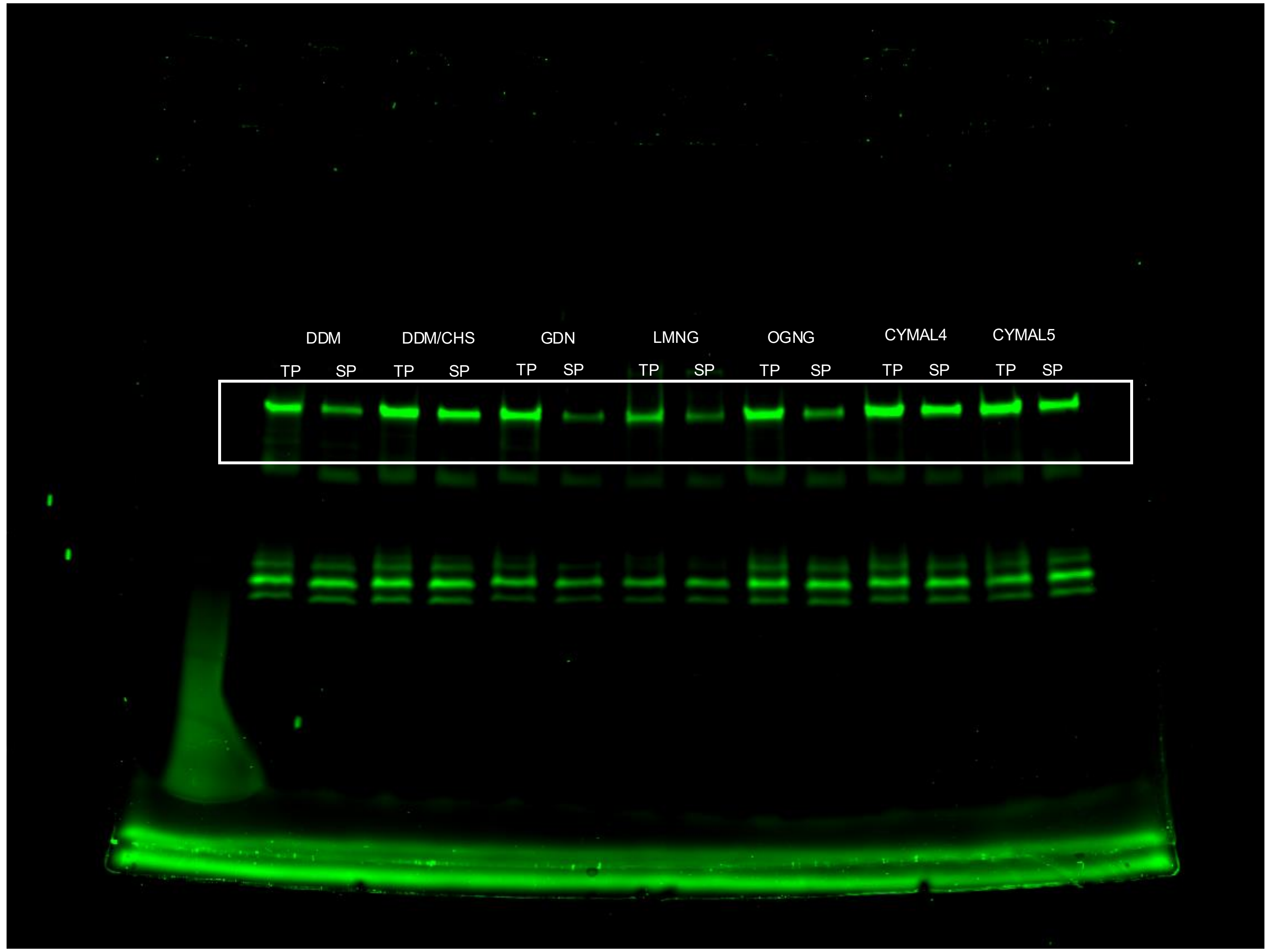

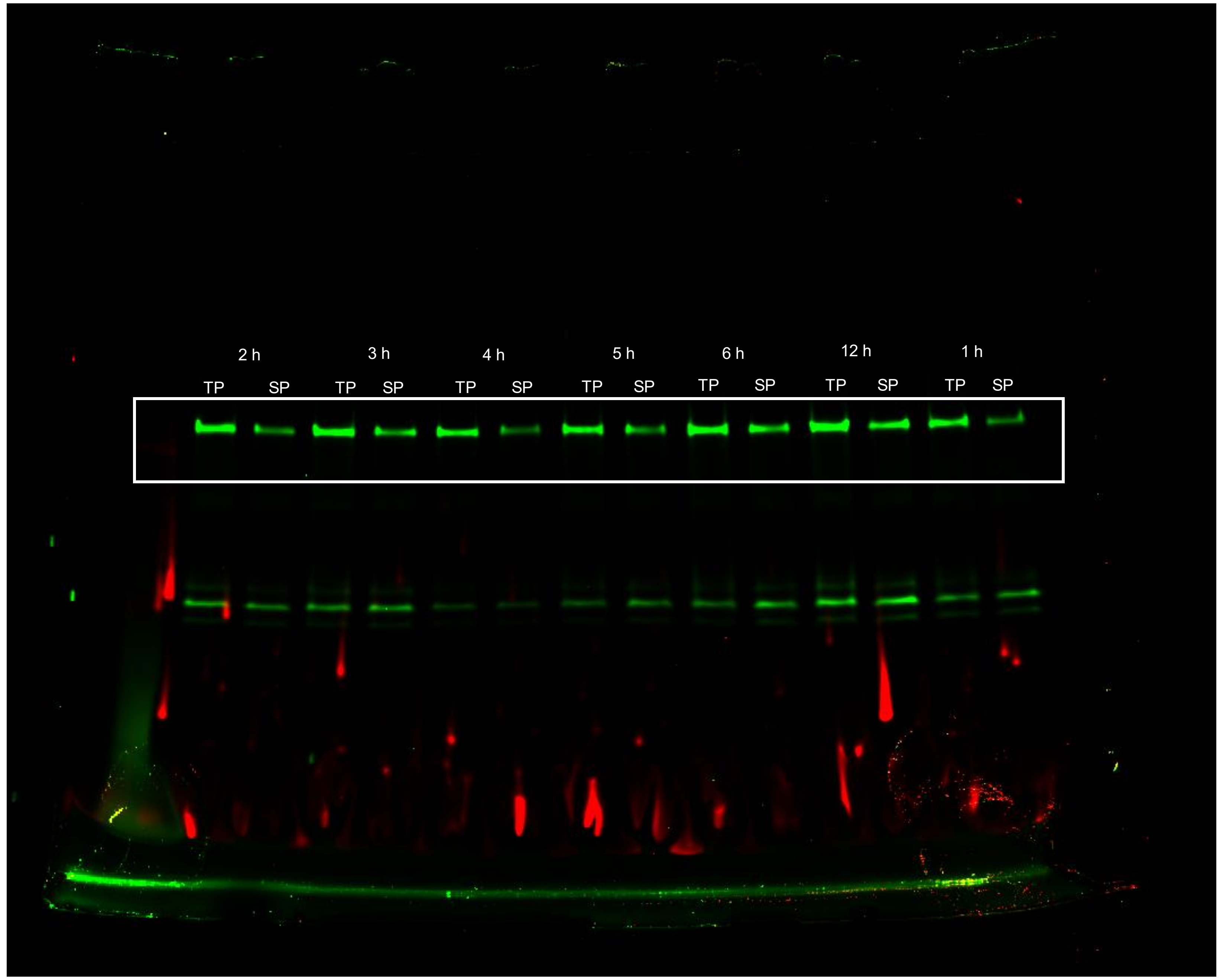

Fig 2. the raw image

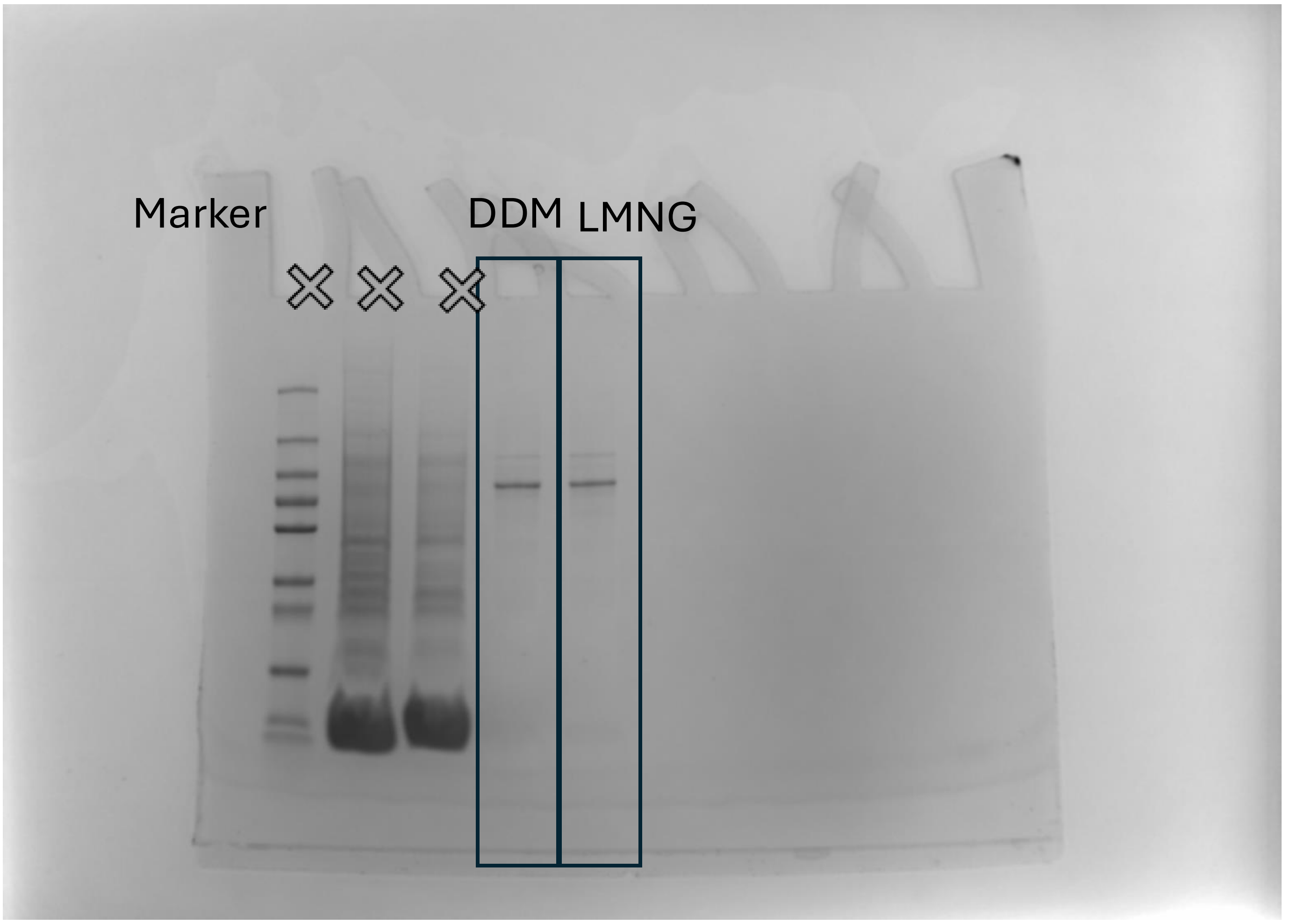

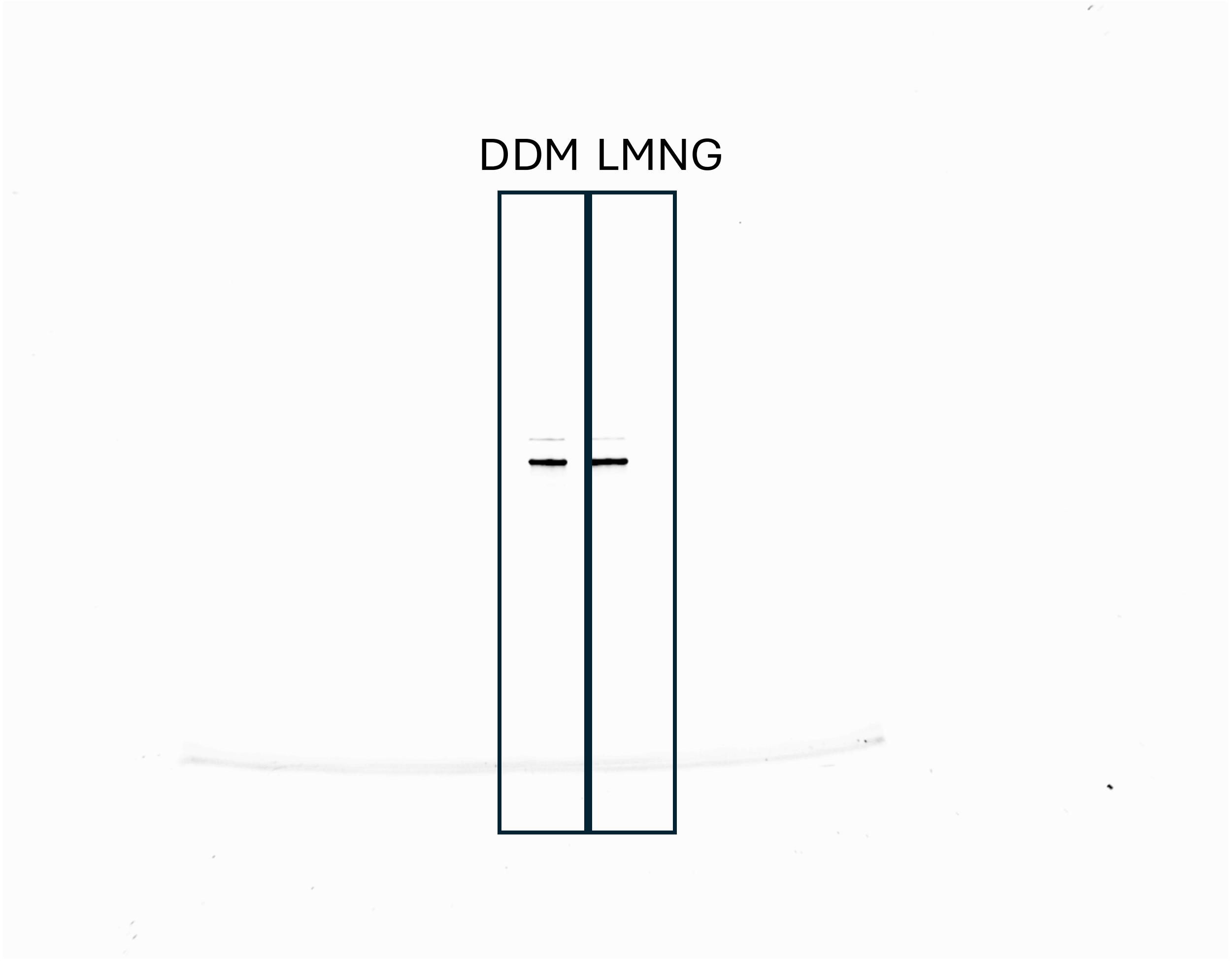

Fig 2 and 4. the raw image

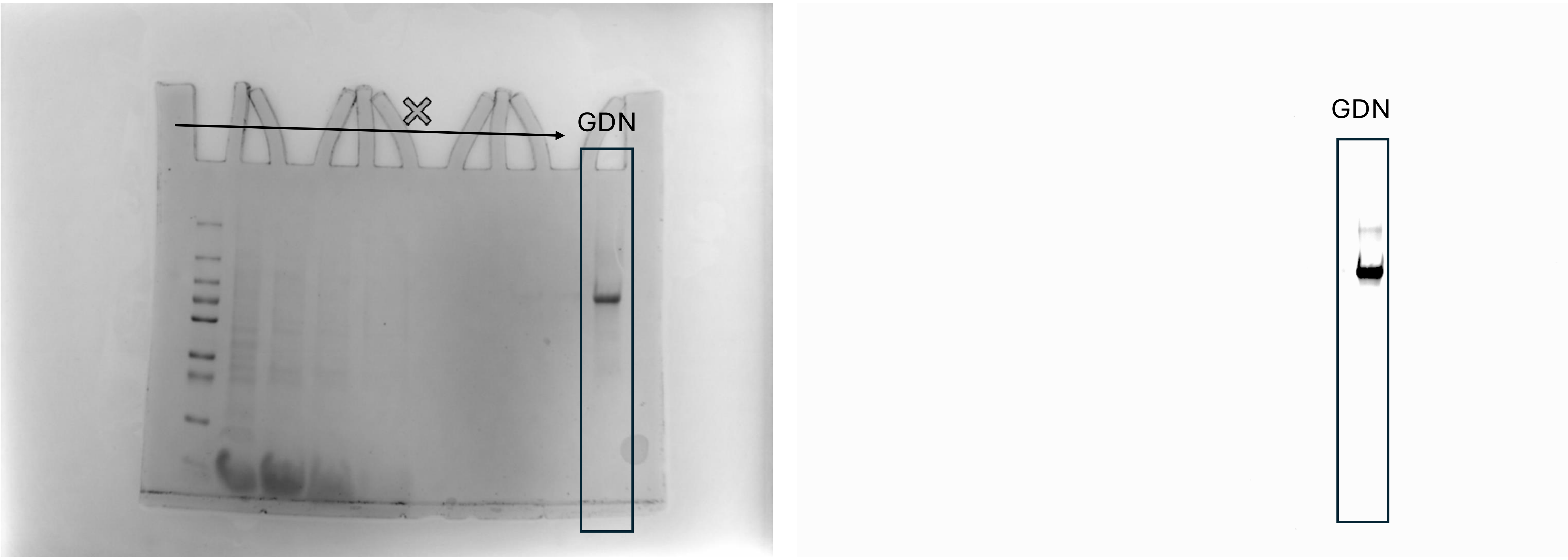

Fig 4. the raw image

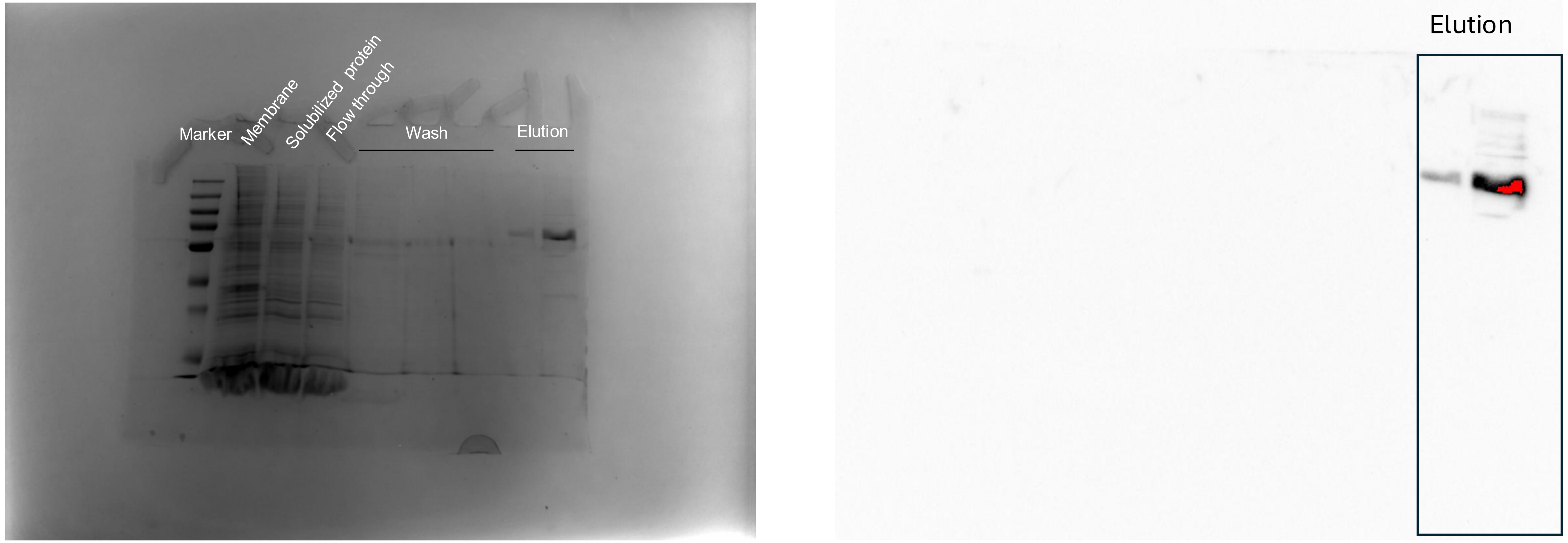

Fig 5. the raw image

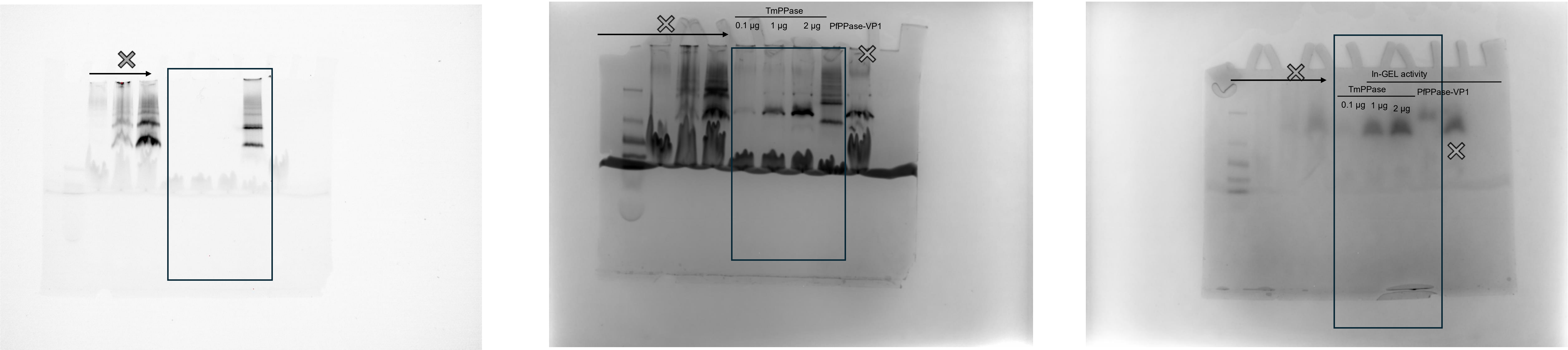

Fig 6. the raw image

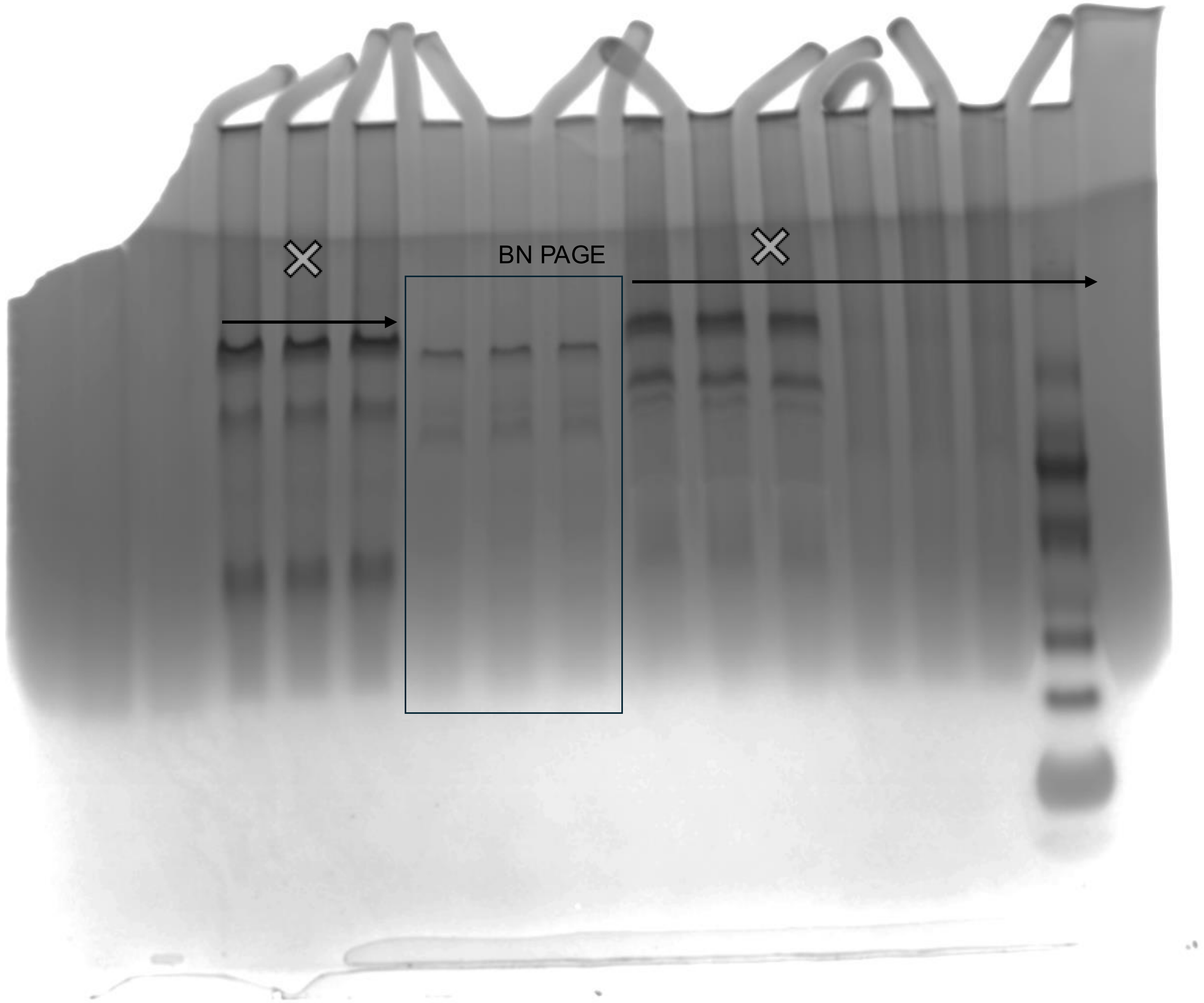

S1.Fig. the raw image

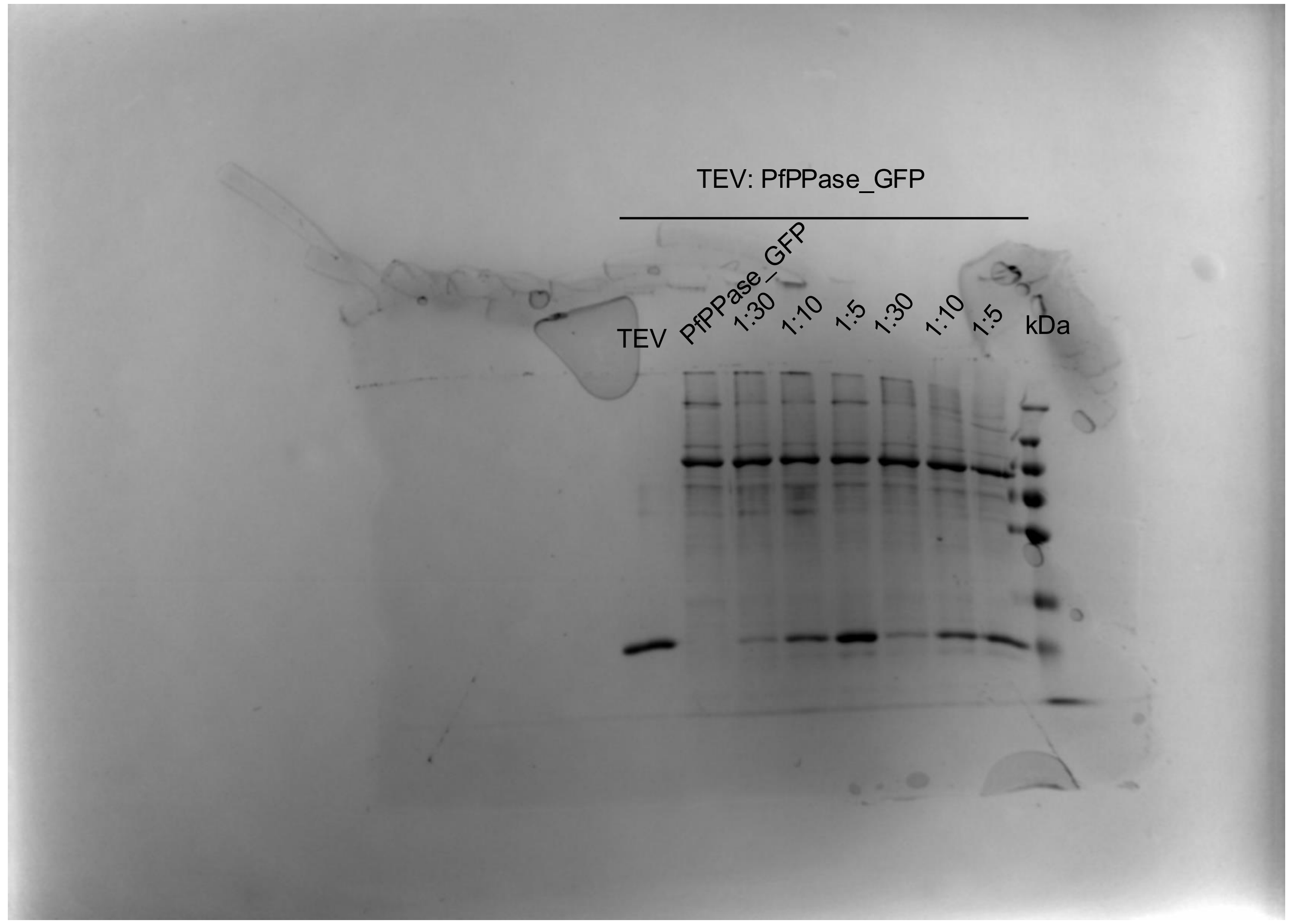

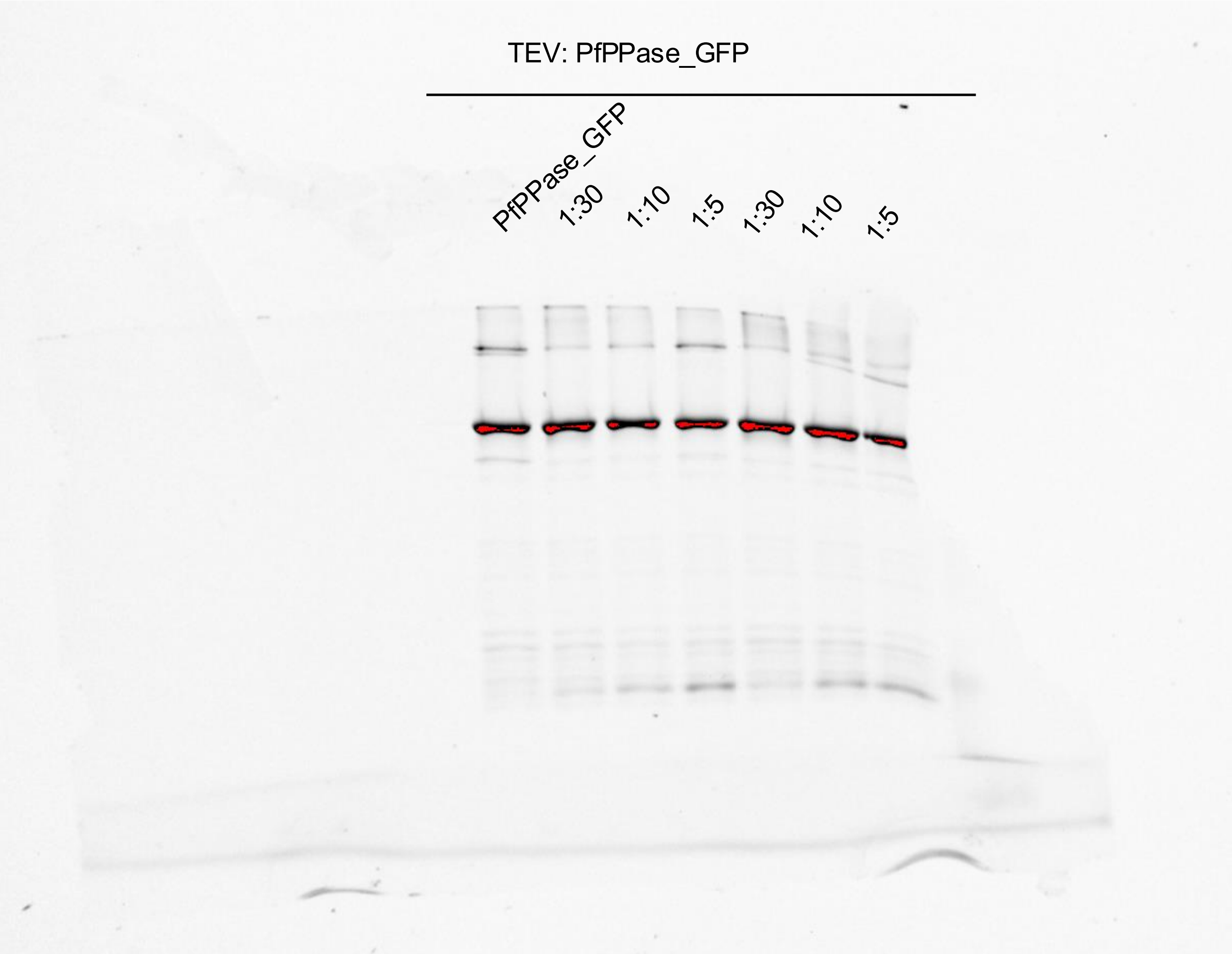

S2. Fig. the raw image

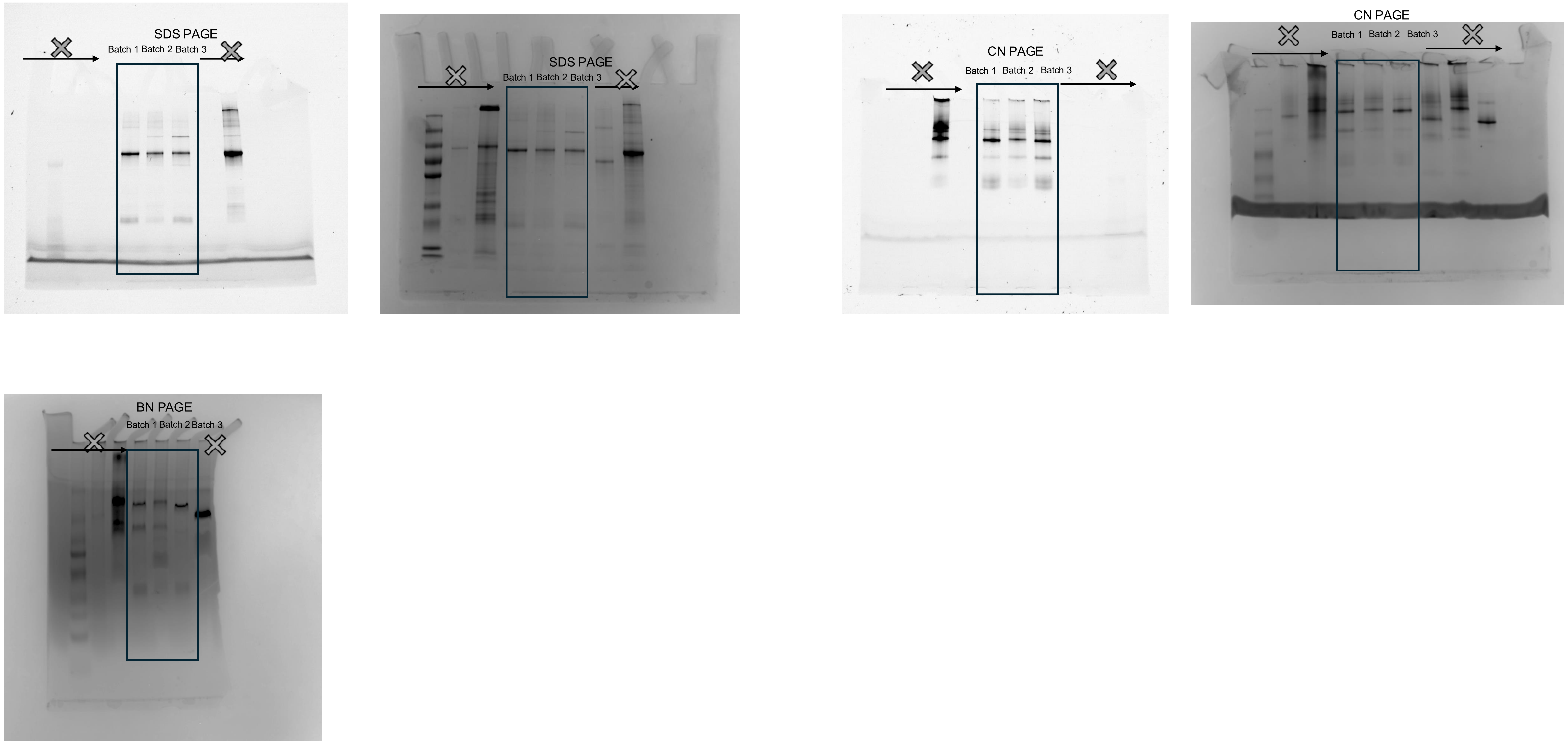

S3.Fig. the raw image
