# Supplementary material for: Expression, purification and preliminary pharmacological characterization of the Plasmodium falciparum membrane-bound pyrophosphatase type 1
Source: PLoS One. 2025 May 27;20(5):e0322756. doi: 10.1371/journal.pone.0322756 (PMC12111632; doi:10.1371/journal.pone.0322756)
Supplement: Attachment — Submitted filename: Response to Reviewers_JL.docx [file pone.0322756.s004.docx]

Reviewer #1: 1. Yes, Hi5 is clearly giving a higher yield. Data Shows expression analysis was done for four days. Did the authors try a plateau or decline in the expression level. To my understanding it would be good practice to optimize the duration of culture to get an optimal yield.

Thank you for your comment. As shown in Figure 1, protein expression plateaued on Day 3 (48 hours post-transfection), with no significant increase observed on Day 4 (72 hours post-transfection). We did not extend the experiment to Day 5, as the cells began to break down and viability declined noticeably (see figure below). This could decrease the quality of the expressed protein. Based on these observations, we selected Day 4 as the optimal harvest time.

2. Yes, statistical tools is used but I am not sure if it is used rigorously. (I think the claim should be made carefully for total yield of desired protein and solubility percentage. Particularly in the case of membrane-bound protein). DDM+CHS solubilized more than other used detergents: I hope the authors checked for the maximum extraction of produced protein. I believe some of the protein remains on the membrane and it cannot be resolved during SDS PAGE. Which may affect the image J based determination of protein yield. Hopefully authors have considered it.

We acknowledge the reviewer concern regarding the determination of solubility percentage.Yes, we took this into careful consideration. To isolate the solubilized protein from the membrane as effectively as possible and to maximize protein yield, we performed ultracentrifugation at 100,000 ×g for 1 hour. A similar method was also used by Liu *et al.,* to screen detergent and calculate protein yield (Liu *et al.,* Methods Mol. Biol.2020). This protocol was consistently applied to all detergents screened, ensuring a fair comparison of their solubilization efficiency. Therefore, we believe the reported percentage of solubilized protein is reliable across all detergents tested.

3. Yes, the data is available
4. I believe the manuscript is in presentable form and written in standard English.

Reviewer #2: Manuscript titled" Expression, purification and preliminary pharmacological characterization of the Plasmodium falciparum membrane-bound pyrophosphatase type 1" by Jianing is a nice piece of work. The work is important and done cleanely. Overall the manuscript looks OK but need minor revision before provisionally accepted for publication. My comments and suggestions to authors are as below.
1) Draft need minor writing editing

Thank you for your input. We have revised the draft based on the reviewers suggestions.
2) In table 1, 2 please add information about tested compound or drugs like CAS no, grade type, purity % etc

We have added additional information about the compounds used in Tables 1 and 2 of the manuscript.

3) In gel image please use white background and bands as black for better visibility

We apologize for the confusion. We have reversed the backgroud from black to white in the GFP fluoresence SDS PAGE images.
4) In Fig 1 A scale bar is missing

Thanks for pointing this out, now we added the scale bar on the Fig 1A.

5) Information about protein purity is missing

Thanks for pointing this out. SDS-PAGE analysis showed a single prominent band corresponding to the expected molecular weight of the target protein, with no detectable impurity bands, suggesting a purity level of >95%. We have included the result in the manuscript.
